# Performance Appraisal and Innovative Behavior in the Digital Era

**DOI:** 10.3389/fpsyg.2019.01659

**Published:** 2019-07-17

**Authors:** Ylenia Curzi, Tommaso Fabbri, Anna Chiara Scapolan, Stefano Boscolo

**Affiliations:** ^1^Marco Biagi Department of Economics, University of Modena and Reggio Emilia, Modena, Italy; ^2^Department of Communication and Economics, University of Modena and Reggio Emilia, Reggio Emilia, Italy

**Keywords:** performance appraisal, innovative work behavior, employee perception, competence-oriented appraisal, informal feedback

## Abstract

In digital competitive environments, organizations’ ability to innovate is more than ever the key to competitive advantage. One way to cope with this increased pressure for innovation is to capitalize on employees’ ability to generate new ideas and use these as building blocks for new and better products, services, and work processes. Individual innovation thus emerges as a key competence required from workers, in turn crucially affecting the way managers make employees contribute to organizational goals and assess their performance. This study draws on the process-based approach to HRM (Bowen and Ostroff, 2004) suggesting that HRM practices may have a signaling effect, to address the following research question: which specific characteristics of performance appraisal are more likely to be perceived as promoting individual innovation at work? To address this issue, we carried out a survey on 865 employees working in large, multinational firms operating in digitalized sectors or industries with the potential to become digitalized. We collected data on the main characteristics of the performance appraisal systems adopted by the firm where respondents work, as perceived by employees themselves. We gathered also data on the respondents’ overall perception that performance appraisal boosts innovative work behavior (IWB). Then, we employed logit analysis to test the relationship between data on performance appraisal systems and data on the effectiveness of performance appraisal as a booster of IWB. Our results reveal that, as compared to informal feedback, formal performance appraisal is more likely to reduce the perception that performance appraisal promotes individual innovation and creativity at work. In addition, we found that in the employees’ perception performance appraisal focused on the achievement of pre-set, quantitative outcomes is more likely to affect positively IWB than appraisal focused on pre-defined skills that employees exhibited performing their work. However, performance assessment focused on the new competences developed by the employees has a perceived positive impact even stronger than result-oriented appraisal. Taken together, these results contribute to advance our understanding of how organizations should evaluate employees in the digitalization era.

## Introduction

In increasingly digital competitive environments, organizations’ ability to innovate is more than ever the key to competitive advantage (e.g., Anderson et al., 2014; Schwarzmüller et al., 2018). Thus, employees at all levels of the organization can help to attain organizational success through their innovative work behavior (IWB), intended as individual extra-role, proactive behavior aimed at generating, disseminating and implementing new ideas in the workplace (Parker et al., 2006).

The key role that employees’ IWB may play in helping organizations to cope with the increased pressure for innovation brought about by digitalization (Colbert et al., 2016; Shanker et al., 2017; Sanz-Valle and Jiménez-Jiménez, 2018) in turn challenges the supervisor–subordinate relationship and reshapes the way managers make their employees contribute to organizational goals. Indeed, several studies on leadership and digitalization (e.g., Chen and Nath, 2008; Schwarzmüller et al., 2018) conceive leadership as a multi-dimensional, overarching construct that captures all kinds of behavior aimed to influence others toward achieving some kind of shared objectives, including both the behavior that seeks to achieve efficiency in work accomplishment (i.e., planning and monitoring) and the behavior that tries to support individual innovation (e.g., empowerment, coaching). In doing so, the above-mentioned studies implicitly consider supervision [defined in terms of giving orders to others since Mintzberg’s (1980) seminal work] and leadership as roles that, to some extent and in some situations, are not mutually exclusive. At the same time, they stress that in the digital age, the supervisor’s job is no longer just to define and distribute tasks and oversee whether they are executed in accordance with rules that strictly predetermine employees’ behavior. Supervisors need to embrace different kinds of behavior, from planning and monitoring to supporting, developing and empowering employees, thus facilitating change processes and encouraging employees’ IWB. This in turn seems to call for a different approach to performance appraisal and management, more focused on fostering individual innovation instead of holding employees accountable for prescribed behavior.

Consistently, an interesting debate on performance management practices has emerged, on the one hand, questioning the effectiveness of traditional performance appraisal (Pulakos and O’Leary, 2011; Pulakos et al., 2015; Cappelli and Tavis, 2016) and, on the other, speculating about changes in performance management and performance appraisal resulting from the digital transformation of work and organizations (Schwarzmüller et al., 2018).

Performance management and particularly performance appraisal is one of the most important HRM practices as it identifies individual responsibilities, objectives and required behavior with the ultimate goal to align employees’ behavior and goals with the company’s strategy (DeNisi and Sonesh, 2011; DeNisi and Murphy, 2017). Thus, in accordance with the signaling theory of HRM and the process-based approach to HRM (Bowen and Ostroff, 2004; Sanders and Yang, 2016), it may be argued that performance appraisal, as perceived by employees, might act as an important signal in digitally transforming organizations subject to an increased pressure for innovation, signaling to employees the importance their companies place on innovative behavior and results and thus promoting individual creativity and innovation at work.

Nevertheless, to the best of our knowledge extant empirical research has neglected to investigate how traditional performance appraisal, as perceived by the employees themselves may promote individual IWB, and thus support organizations to meet the demand for creativity and innovation in the digitalization era. In fact, although a growing body of research has recently emerged within the process-based approach to HRM, particularly focusing on the perceived relationships between HRM practices and IWB (Bednall et al., 2014; Bos-Nehles and Veenendaal, 2017; Escribá-Carda et al., 2017; Sanders et al., 2018), it has not yet addressed such a relationship in the context of digitalization. Similarly, extant studies on digitalization and HRM have so far devoted attention to electronic performance management systems, focusing only on how computerized and digital performance measurement and feedback may affect the efficiency (cost and times) of the performance management process and employees’ reactions to performance appraisal (Stone et al., 2015). Only some scholars have suggested that under conditions of digital work leaders and performance management systems should display a higher output orientation (i.e., a stronger focus on the achievement of objective goals rather than on pre-set behavior, time spent at the office and long working hours) and greater concern for personnel development so as to help employees to meet the competence requirements of digital work (Staples et al., 1999; Chen and Nath, 2005, 2008; Nijp et al., 2016). However, “research on this topic appears as practically still in its infancy” (Schwarzmüller et al., 2018, p. 116).

Therefore, our research aims at filling this gap by addressing the following question: which specific characteristics of traditional performance appraisal – in terms of “how” the appraisal may be conducted and “what” may be evaluated – are more likely to be perceived as promoting individual innovation at work?

In order to address such research question, we carried out a survey on 865 employees working in large, multinational firms located in Italy and operating in digitalized sectors or in industries with the potential to become digitalized (McKinsey Global Institute, 2016).

## Theoretical Framework

In order to investigate whether and how performance appraisal may enhance IWB, we built on the HRM studies which adopt a process-based approach (Bowen and Ostroff, 2004; Sanders and Yang, 2016).

This approach suggests interpreting HRM practices as messages that organizations send to their leaders and employees to inform about which results and behavior (e.g., IWB) are expected, supported, encouraged and eventually rewarded (Chang, 2005; Bednall et al., 2014; Bos-Nehles and Veenendaal, 2017; Escribá-Carda et al., 2017; Sanders et al., 2018). In accordance to this signaling perspective, HRM practices are able to elicit the desired behavior and attitudes particularly when employees perceive HRM practices as understandable (i.e., they are not ambiguous), consistent (they indeed do what they are intended to do, e.g., they promote IWB), and consensual (employees agree about the perception of those HRM practices).

Consistently, the basic function of performance appraisal is identifying and communicating individual responsibilities, expected objectives, required behavior and competences, ensuring the alignment between individuals’ behavior and goals and the organization’s strategic goals (DeNisi and Sonesh, 2011; DeNisi and Murphy, 2017). Thus, performance appraisal may stand out in the realm of HRM practices that organizations aimed at coping with the strategic challenges of digitalization may use in support of leaders to encourage employees’ IWB.

More specifically, a first characteristic of performance appraisal which may have a potential signaling effect in terms of promotion of IWB, is the formality of the appraisal system, i.e., the extent to which leaders give employees feedback by means of formal tools and procedures, at prescribed times (e.g., traditional annual or bi-annual appraisal reviews conducted through a standard rating form). This characteristic is widely discussed in the lively debate on performance appraisal, where critics question this feature and point to the increasing number of leading corporations that are substituting their traditional formal performance appraisal system with informal feedback basically because they deem the former inappropriate to help leaders to support employees in learning new things and being creative and innovative (Pulakos and O’Leary, 2011; Pulakos et al., 2015; Cappelli and Tavis, 2016). There are indeed some studies showing a weak positive or even a negative relationship between formal HRM practices as perceived by the employees and IWB (Bednall et al., 2014; Sanders and Yang, 2016). However, there is also evidence of a positive relationship between formal appraisal and the generation and use of new ideas at work (Shipton et al., 2005, 2006; Gorbatov and Lane, 2018). Such evidence echoes recent studies which have pointed out that structuring HRM processes is perceived as related positively to employees’ creativity (Binyamin and Carmeli, 2010; Sanders et al., 2018). Moreover, it is consistent with Bowen and Ostroff’s (2004) conceptualization, which suggests that formal HRM practices may increase the understandability and transparency of such practices thereby raising the likelihood that these practices will have the desired effect, i.e., in our case, promoting IWB.

As a result, we formulate our first research hypothesis as follows:

H1. In the employees’ perception, formal performance appraisal relates positively to IWB.

A second important characteristic of performance appraisal regards the appraisal criteria. About this, useful insights are provided by studies which underlined the positive influence of job autonomy on IWB (Criscuolo et al., 2014; Bos-Nehles et al., 2017) and studies which argued that the leader’s behavior should emphasize delegation (de Jong and Den Hartog, 2007). These studies in fact suggest that performance appraisal focused on the outcomes achieved by employees (as compared to performance targets) rather than on the extent to which they conform to the behavior that has proven to be effective in the past is more likely to foster IWB. This insight is consistent with the scholars’ recommendation that, in accordance with the new role required from leaders by the digital transformation of work, managers should pre-set specific (i.e., clear and measurable) performance goals, allowing employees to be creative in the pursuit of these targets (Staples et al., 1999; Chen and Nath, 2005, 2008; Nijp et al., 2016; Schwarzmüller et al., 2018). Furthermore, there is an extensive body of research pointing out that result-oriented appraisal positively affects employees’ job satisfaction, thereby increasing their commitment to align to the behavior the organization wishes to encourage (Kampkkötter, 2016). In particular, management by objectives (hereinafter MBO), which combine performance-related pay and result-oriented appraisal, was found to be one of the key determinants of digital workers’ job satisfaction (Konradt et al., 2003). Drawing on this literature, it is reasonable to expect that employees will be more likely to perceive performance appraisal as a practice intended to promote IWB when it is result-oriented, i.e., focused on the achievement of performance targets.

On the other hand, there are several studies investigating the impact of pay-for-performance schemes which have found no or even a negative relationship to IWB (Shipton et al., 2005, 2006; Bos-Nehles and Veenendaal, 2017; Sanders et al., 2018). Since performance-related pay is frequently associated to result-oriented performance appraisal (like for instance MBO), these studies seem to question the existence of a positive relationship between this kind of appraisal and IWB. Indeed, it has been argued that result-oriented pay (and appraisal) tends to focus the individuals’ attention on the achievement of pre-set short-term performance goals, thereby signaling to employees that it is better to direct efforts toward the fine-tuning of work methods they have already tried in the past rather than exploring radically new ways of doing work. As a result, scholars suggested, but did not test empirically, that organizations wishing to encourage IWB should design appraisal systems focused on knowledge, skills and competences as a key to creativity and innovation (Sanders et al., 2018). Thus, a widespread practice is asking leaders to rate the skills their employees have exhibited during the execution of their tasks (Pulakos and O’Leary, 2011), by comparing them to pre-set competences (basically, the skills included in the role description). Moreover, the predefinition of skills becomes usual when tasks uncertainty increases (Mintzberg, 1980), preventing leaders from defining rules and procedures that strictly predetermine how to execute tasks and leaving employees some leeway to decide how to perform work. Consequently, it is likely that employees perceive exhibited skill-oriented appraisal as a practice aimed at encouraging their IWB. Furthermore, it has been underlined that competence-oriented performance appraisal should have also a development purpose (Shalley and Perry-Smith, 2001) because, due to this purpose, employees will be more likely to perceive appraisal as a practice specifically designed to meet their basic need to feel competent and autonomous, also in exploring new ways of doing things (Gagné and Deci, 2005). This suggests that performance appraisal that focuses on the new competences developed by employees while performing their work are more likely to be perceived as a booster of IWB, particularly in digital work settings where leaders are asked to help employees to meet increased demand for creativity and innovation (Schwarzmüller et al., 2018).

Drawing on the above mixed empirical evidence, we formulate our second hypothesis as follows:

H2a. In the employees’ perception, result-oriented appraisal relates positively to IWB.
H2b. In the employees’ perception, exhibited skill-oriented appraisal relates positively to IWB.
H2c. In the employees’ perception, new competence-oriented appraisal relates positively to IWB.

Finally, since performance appraisal intended as a process begins with goal setting, another characteristic that may be key to the signaling effect of performance appraisal in terms of promotion of IWB is employees’ involvement in goal setting. Regarding this, research, particularly in the field of participative leadership, identified that the involvement of employees themselves in setting the goals they are expected to achieve in their work has positive influence on IWB (de Jong and Den Hartog, 2007). Moreover, scholars suggested that employees’ involvement in setting the target objectives to be included in MBO increases the effectiveness of this result-oriented pay (and appraisal) system (e.g., McConkie, 1982). Finally, studies conducted during earlier stages of the digital transformation argued that digital work would not only call for new performance appraisal systems focused more on the results achieved by the employees rather than on time spent at the office, but also for more employees’ involvement in setting work goals and priorities (Staples et al., 1999). Thus, it is reasonable to expect that result-oriented performance appraisal will be more likely to be perceived as conducive to IWB when it is implemented together with employees’ involvement in goal setting.

Since goals, rather than being performance-oriented, can be learning-oriented (Locke and Latham, 2002), we suggest that also exhibited skill-oriented and new competence-oriented appraisal systems may involve employees in goal setting, thus strengthening the employees’ perception that such systems promote IWB. Consistently, we formulate our third hypothesis as follows:

H3a. In the employees’ perception, the employees’ involvement in goal setting will positively moderate the strength of the relationship between result-oriented appraisal and IWB, so that this relationship will be stronger when employees’ involvement in goal setting is higher.
H3b. In the employees’ perception, the employees’ involvement in goal setting will positively moderate the strength of the relationship between exhibited skill-oriented appraisal and IWB, so that this relationship will be stronger when employees’ involvement in goal setting is higher.
H3c. In the employees’ perception, the employees’ involvement in goal setting will positively moderate the strength of the relationship between new-competence oriented appraisal and IWB, so that this relationship will be stronger when employees’ involvement in goal setting is higher.

## Materials and Methods

### Procedure and Sample

We collected data between December 2017 and the end of January 2018 by administering an on-line survey to a sample of 1250 Italian employees. We used a non-probabilistic sampling method, namely convenience sampling. We collected 865 usable questionnaires. Around 83% of respondents were male, nearly 57% were less than 45 years old and had a master degree or above. Nearly 46% of respondents had a company’s tenure of more than 10 years; 45% held managerial positions and only 14% worked in the research and development department.

Table 1 shows the percentage distribution of respondents depending on the main characteristics of the companies where they work, i.e., sector, the sector’s level of digitalization,^1^ size, geographical location and nationality of the company. Regarding the size, the most part of respondents (about 70%) work in companies with more than 250 employees. This is remarkable since literature has identified the large size of the business as a key determinant of the decision to adopt complex HRM practices, including performance appraisal (Bayo-Moriones et al., 2013) as well as of the decision to embrace digital transformation (McKinsey Global Institute, 2016).

**TABLE 1:** Characteristics of the companies where the respondents work.

	**%**
**Manufacturing sectors**	**45.7**
**Service sectors**	**52.4**
**Other sectors** (i.e., agriculture and construction)	**1.9**
**Digitalized sectors**	**49**
ICT	22.4
Media	1.8
Finance and insurance	13.9
Professional services	10.9
**Sectors with the potential to become digitalized**	**26**
Agriculture	0.6
Basic goods manufacturing	1.6
Chemicals and pharmaceuticals	7.2
Construction	1.4
Electricity, gas, steam and air conditioning supply. Water supply; sewerage, waste management and remediation activities	5.7
Entertainment and recreation	0.3
Health care and social assistance	2.0
Hospitality	0.9
Personal services	0.9
Retail trade	2.6
Transportation and storage	2.8
**Lagging sectors**	**25**
Business support activities	2.8
Food and beverage	3.5
Motor vehicles and other transport equipment	8.2
Machinery and equipment; fabricated metal products	4.0
Other non-metallic mineral products	0.8
Paper	0.8
Rubber and plastic	2.3
Textile	2.2
Wood and furniture	0.4
**Large firms (with more than 250 employees)**	**69**
**Northern Italy firms**	**70**
**Multinational companies**	**71**
Italian company with subsidiaries abroad	36.8
Italian branch of a foreign company	34.2

Table 2 presents the percentage distribution of answers concerning the main characteristics of the performance appraisal systems used in the companies where respondents work, i.e., the formality of performance appraisal the appraisal criteria, the employees’ involvement in goal setting, as well as the feedback process and the link between performance appraisal and remuneration. In accordance to Bowen and Ostroff (2004) and many other studies using the signaling theory and the process-based approach to HRM, we used employees rather than the HR department or supervisors/managers as the primary information source of the main features of performance appraisal systems.

**TABLE 2:** Characteristics of the performance appraisal systems used in the companies where the respondents work.

	**%**
**How the employees’ performance is evaluated**	
By formal appraisal systems and company’s procedures	68.7
Discretionary judgment without an appraisal form	19.8
Supervisor’s informal appraisal at the employee’s request	11.6
**Performance appraisal criteria^2^**	
Achievement of formal individual performance targets	69.6
The results achieved by the company	38.0
The employee’s behavior during the past year	20.8
Accomplishment of all assigned tasks	26.8
The skills the employee has exhibited during the execution of his/her tasks	45.8
The new competences developed by the employee as s/he performs his/her work	17.3
The employee’s personality and personal characteristics	20.3
The employee’s goodwill and active involvement	35.7
Workplace attendance (presenteeism)	11.7
Having performed additional hours of work/overtime	6.6
The intensity with which the employee works	7.3
**Employees’ involvement in the appraisal process**	
Employee’s involvement in goal setting	54
**Whether employees receive feedback information about their appraisal ratings and how**	
No, employees receive no feedback information	10.9
Yes, they receive formal feedback	58.4
Yes, they receive informal feedback by their immediate boss or by the top management	24.2
Yes, they receive feedback information at their request	6.6
**Whether employees’ performance appraisal is linked to remuneration**	
Yes, in the form of merit increases	10.9
Yes, in the form of cash bonuses based on the achievement of individual targets	27.7
Yes, in the form of cash bonuses based on the achievement of overall organizational objectives	11.0
Yes, in the form of cash bonuses given at the discretion of the supervisor or the ownership	10.1
Yes, in the form of cash bonuses based on non-transparent criteria of distribution	2.2
Yes, in the form of non-monetary reward (e.g., benefits and perks etc.)	0.5
No, employees’ performance appraisal is not linked to any kind of reward	37.6

*^2^A multiple-choice question was used to collect information about which appraisal criteria were used in the company to assess the employee’s performance with the possibility for the respondents to select three modalities of response. Therefore, the percentage frequency of each modality shows how many respondents indicated the corresponding appraisal criterion out of 100 who answered the question.*

### Measures

#### Independent Variables

##### Formal performance appraisal

It was measured using a dummy variable with 1 = the employee’s performance is evaluated by formal appraisal systems/procedures and 0 = the employee’s performance appraisal is informal (i.e., discretionary judgment without an appraisal form or supervisor’s informal appraisal at the employee’s request).

##### Result-oriented appraisal

It was measured using a dummy variable with 1 = performance appraisal focuses on the achievement of formal, individual performance targets and 0 = performance appraisal is not focused on the achievement of performance targets.

##### Exhibited skill-oriented appraisal

It was measured using a dummy variable with 1 = performance appraisal focuses on skills exhibited by the employee and 0 = performance appraisal does not focus on exhibited skills.

##### New competence-oriented appraisal

It was measured using a dummy variable with 1 = performance appraisal focuses on the new competences developed by the employee and 0 = performance appraisal does not focus on new developed competences.

##### Employees’ involvement in goal setting

It was measured using a dummy variable with 1 = the employee is involved in the formulation of the goals s/he will be evaluated for and 0 = s/he is not involved.

#### Dependent Variable

##### IWB

Respondents were asked whether and how performance appraisal affects the generation and implementation of creative ideas in the workplace, with the following possible answers: yes, positively; yes, negatively; no influence. This categorical variable was then transformed into a dummy variable with 1 = perceived positive influence and 0 = perceived negative influence or no influence.

#### Control Variables

We also controlled for the following employees’ and organizational characteristics: job position, age, educational level, organizational tenure, gender, company’s department (i.e., R&D), firm’s size, sector (manufacturing vs. others, the latter including services as well as agriculture and construction), the sector’s level of digitalization (i.e., digitalized sectors and sectors with the potential to become digitalized), geographical location, and whether the firm is or belongs to a multinational.

### Data Analysis

We used Stata 14.1 SE (StataCorp, College Station, TX) to conduct statistical analyses. First, the Kruskal–Wallis *H* test (Kruskal and Wallis, 1952) was conducted to assess whether there were statistically significant differences in the independent and dependent variables depending on the company’s sector (i.e., manufacturing vs. other sectors) and the sector’s level of digitalization. Regarding the latter, we also conducted the Dunn’s pairwise z test with the Bonferroni’s correction for multiple pairwise comparisons (Dunn, 1961, 1964; Dinno, 2015) to identify which sectors differ. Then, to test the hypotheses about main and moderating effects, also checking the influence of the control variables, we conducted logit analysis (Cameron and Trivedi, 2018). Precisely, we tested for non-linear associations between formal performance appraisal, the three performance appraisal criteria, employees’ involvement in goal setting and the perceived effectiveness of performance appraisal as a booster of IWB as well as for the interaction effects between the three performance appraisal criteria and the employees’ involvement in goal setting. We calculated the variance inflation factor (VIF) of each regressor in order to check for multi-collinearity (Cohen et al., 2003). In addition, given the S-shaped curve fitted by logistic regression models, we calculated the average marginal effect of each regression in order to estimate its influence on the dependent variable.

## Results

The Kruskal–Wallis one-way ANOVA test shows statistically significant differences regarding the perception of performance appraisal as a booster of IWB, the result-oriented performance appraisal and the employees’ involvement in goal setting between employees in manufacturing and those in other sectors (see Table 3). More specifically, the mean ranks show that more employees in manufacturing perceive performance appraisal as a booster of IWB, are subject to a performance appraisal focused on results and are involved in setting goals than in other sectors.

**TABLE 3:** Kruskal–Wallis *H* test using sector as grouping variable.

**Variables**	**χ^2^(1)**	**Mean ranks**	
	
	***p*-Value**	**Manufacturing (*N* = 395)**	**Other Sectors (services, agriculture and construction) (*N* = 470)**
Perception of performance appraisal as a booster of IWB	3.358 (*p* = 0.0669)	447.70	420.65
Result-oriented performance appraisal	3.219 (*p* = 0.0728)	446.25	421.87
Employees’ involvement in goal setting	5.900 (*p* = 0.0151)	452.43	416.67
			

Similarly, the Kruskal–Wallis test shows statistically significant differences regarding the perception that performance appraisal boosts IWB and the formality of the appraisal process between employees in digitalized sectors, those in sectors with the potential to become digitalized and employees in lagging sectors (see Table 4).

**TABLE 4:** Kruskal–Wallis *H* test using sector’s level of digitalization as grouping variable.

	**χ^2^(2)**	**Mean ranks**		
	
	***p*-Value**	**Lagging sectors (*N* = 215)**	**Sectors with the potential to become digitalized (*N* = 225)**	**Digitalized sectors (*N* = 425)**
Perception of performance appraisal as a booster of IWB	7.777 (*p* = 0.0205)	458.44	447.36	412.53
Formal performance appraisal	4.887 (*p* = 0.0869)	411.59	426.26	447.40

More specifically, according to the mean ranks and Dunn-Bonferroni’s tests (see Table 5), more employees in sectors with the potential to become digitalized and in lagging sectors perceive performance appraisal as a booster of IWB than in digitalized sectors. Moreover, more employees in digitalized sectors are subject to formal performance appraisal than in lagging sectors.

**TABLE 5:** Dunn’s test with Bonferroni’s correction for multiple pairwise comparisons using sector’s level of digitalization as grouping variable.

	**Perception of performance appraisal as a booster of IWB**		**Formal performance appraisal**	
	**Lagging sectors**	**Sectors with the potential to become digitalized**	**Lagging sectors**	**Sectors with the potential to become digitalized**
Sectors with the potential to become digitalized	*z* = 0.538 (*p* = 0.8863)		*z* = −0.766 (*p* = 0.6656)	
Digitalized sectors	*z* = 2.537 (*p* = 0.0168)	*z* = 1.953 (*p* = 0.0761)	*z* = −2.132 (*p* = 0.0496)	*z* = −1.278 (*p* = 0.3020)

Since the above analyses suggest that the sector and the sector’s level of digitalization might have an influence on the dependent variable and some independent variables, we decided to include manufacturing, sectors with the potential to become digitalized and digitalized sectors as control variables (all measured as dummies) in the models we estimated to test our main hypotheses.

The mean values, standard deviations and correlations between all variables are presented in Table 6.

**TABLE 6:** Means, standard deviations and correlations.

**VARIABLES**	**Mean**	**Std. dev.**	**1**	**2**	**3**	**4**	**5**	**6**	**7**	**8**	**9**	**10**	**11**	**12**	**13**	**14**	**15**	**16**	**17**	**18**	**19**	**20**	**21**	**22**	**23**	**24**	**25**	**26**
1. Perception of performance appraisal as a booster of IWB	0.52	0.50																										
2. Job position (manager)	0.45	0.50	0.06																									
3. Firm size 51–250	0.19	0.40	0.10	–0.05																								
4. Firm size 251–1000	0.22	0.42	–0.02	–0.01	–0.26																							
5. Firm size > 1000	0.47	0.50	–0.07	0.07	–0.46	–0.50																						
6. Location (North)	0.71	0.46	0.00	0.00	0.08	–0.02	–0.08																					
7. Age 35–44	0.35	0.48	0.04	–0.08	0.05	–0.04	0.00	0.00																				
8. Age 45–54	0.30	0.46	–0.10	0.22	–0.02	–0.01	0.04	0.04	–0.49																			
9. Age > 54	0.12	0.33	0.05	0.25	–0.04	0.06	–0.03	–0.07	–0.28	–0.25																		
10. Tenure ≤ 5	0.36	0.48	0.07	–0.20	0.11	–0.06	–0.13	0.07	–0.07	–0.23	–0.22																	
11. Tenure > 10	0.46	0.50	–0.05	0.23	–0.09	0.03	0.12	–0.08	–0.11	0.30	0.29	–0.69																
12. Education_low	0.29	0.45	0.05	–0.02	0.02	0.04	–0.04	0.00	–0.16	0.12	0.23	–0.18	0.23															
13. Education_medium	0.14	0.35	–0.03	–0.22	0.08	–0.01	–0.07	0.01	0.02	–0.10	–0.09	0.15	–0.17	–0.26														
14. R&S	0.14	0.35	–0.01	0.00	0.03	–0.02	0.02	0.05	–0.05	0.07	–0.01	–0.04	0.04	0.00	–0.09													
15. Manufacturing sectors	0.46	0.50	0.06	0.03	0.04	0.03	0.00	0.06	–0.04	0.07	0.05	–0.01	0.03	0.05	–0.06	0.14												
16. Sectors with the potential to become digitalized	0.26	0.44	0.04	0.03	0.06	–0.02	–0.01	0.02	0.00	–0.03	0.09	–0.03	0.03	0.03	–0.01	–0.09	0.12											
17. Digitalized sectors	0.49	0.50	–0.09	–0.05	–0.10	–0.02	0.08	–0.11	0.00	–0.01	–0.09	0.02	0.00	–0.07	0.05	0.00	–0.53	–0.58										
18. Multinational firm	0.71	0.45	–0.03	0.10	–0.14	0.02	0.28	0.00	0.05	–0.06	–0.04	–0.07	0.01	–0.02	–0.07	0.03	0.18	–0.06	–0.01									
19. Gender	0.17	0.38	–0.03	–0.10	0.05	0.04	–0.08	0.02	0.03	–0.04	–0.09	0.04	–0.05	–0.05	0.00	–0.06	–0.11	–0.01	0.01	0.03								
20. Formal performance appraisal	0.69	0.46	–0.06	0.13	–0.20	0.01	0.30	–0.02	–0.06	0.06	0.07	–0.14	0.14	–0.04	–0.08	0.03	0.02	–0.02	0.07	0.25	0.02							
21. Result-oriented appraisal	0.70	0.46	0.10	0.14	0.00	–0.05	0.09	0.02	0.02	–0.01	0.05	–0.05	0.04	–0.02	0.00	–0.02	0.06	0.02	–0.05	0.14	–0.02	0.32						
22. Exhibited skill-oriented appraisal	0.46	0.50	0.08	0.03	0.05	–0.06	0.01	0.02	0.00	–0.01	0.07	–0.04	0.01	0.06	0.02	0.00	–0.01	–0.01	–0.03	0.01	–0.03	0.05	–0.05					
23. New competence-oriented appraisal	0.17	0.38	0.11	0.00	0.00	–0.01	–0.01	0.04	0.07	–0.09	–0.02	0.05	–0.02	0.00	0.08	0.02	–0.05	–0.01	0.06	0.02	0.03	0.07	0.04	–0.03				
24. Employees’ involvement in goal setting	0.54	0.50	0.20	0.18	0.00	–0.07	0.06	0.04	–0.01	0.02	0.05	–0.05	0.05	0.00	–0.03	0.07	0.08	–0.01	–0.03	0.13	–0.06	0.18	0.32	0.03	0.13			
25. Employees’ involvement in goal setting × result-oriented appraisal	0.07	0.22	0.02	–0.01	0.00	–0.01	0.00	0.06	–0.01	0.02	–0.01	0.01	0.00	–0.05	0.00	–0.06	0.03	–0.01	–0.01	–0.04	0.02	–0.11	–0.28	–0.01	–0.05	–0.05		
26. Employees’ involvement in goal setting × exhibited skill-oriented appraisal	0.01	0.25	0.00	–0.02	–0.03	0.03	0.01	0.02	–0.04	0.04	–0.06	–0.01	–0.01	0.05	0.01	–0.06	0.05	0.01	–0.05	0.03	0.01	0.01	–0.01	0.00	–0.08	0.00	–0.06	
27. Employees’ involvement in goal setting × new competence-oriented appraisal	0.03	0.18	–0.04	–0.01	0.03	–0.01	0.00	0.01	0.01	0.01	–0.01	–0.02	0.00	0.03	0.02	–0.02	0.02	0.04	–0.04	–0.01	0.04	0.03	–0.05	–0.08	0.24	–0.02	0.01	–0.03

Table 7 shows the results of the logistic regression models, specifically the average marginal effect of each regressor. The mean values of the VIF, which are considerably lower than 10, confirm that there is not multi-collinearity between the regressors included in the models presented.

**TABLE 7:** Average marginal effects.

**Variables**	**Model 1**	**Model 2**	**Model 3**	**Model 4**
Job position (manager)	0.102^∗∗∗^	0.083^∗∗^	0.060	0.058
Firm size 51–250	0.088	0.084	0.095	0.097
Firm size 251–1000	–0.026	0.001	0.025	0.024
Firm size > 1000	–0.029	–0.007	0.010	0.008
Location (North)	–0.020	–0.028	–0.032	–0.035
Age 35–44	–0.006	–0.015	–0.010	–0.008
Age 45–54	–0.141^∗∗^	–0.123^∗∗^	–0.116^∗∗^	–0.114^∗∗^
Age > 54	–0.032	–0.027	–0.022	–0.019
Tenure ≤ 5	0.053	0.049	0.054	0.052
Tenure > 10	–0.008	–0.008	–0.008	–0.011
Education_low	0.072^*^	0.054	0.053	0.056
Education_medium	–0.033	–0.065	–0.065	–0.065
R&S	–0.016	–0.016	–0.031	–0.027
Manufacturing sectors	0.029	0.035	0.025	0.023
Sectors with the potential to become digitalized	–0.025	–0.019	–0.017	–0.015
Digitalized sectors	–0.076	–0.065	–0.066	–0.067
Multinational firm	–0.031	–0.036	–0.050	–0.050
Gender	–0.038	–0.033	–0.024	–0.023
Formal performance appraisal		–0.089^∗∗^	–0.102^∗∗^	–0.098^∗∗^
Result-oriented appraisal		0.124^∗∗∗^	0.070^*^	0.081^∗∗^
Exhibited skill-oriented appraisal		0.089^∗∗∗^	0.082^∗∗^	0.079^∗∗^
New competence-oriented appraisal		0.152^∗∗∗^	0.123^∗∗∗^	0.138^∗∗∗^
Employees’ involvement in goal setting			0.178^∗∗∗^	0.174^∗∗∗^
Employees’ involvement in goal setting × result-oriented appraisal				0.097
Employees’ involvement in goal setting × exhibited skill-oriented appraisal				0.029
Employees’ involvement in goal setting × new competence-oriented appraisal				–0.126
Observations	865	865	865	865
Mean VIF	2.70	2.77	2.78	2.62
AUC	0.6226	0.6568	0.6816	0.6865
Pseudo *R*^2^	0.0330	0.0566	0.0785	0.0816
Log likelihood	-579.2	-565.1	-552.0	-550.1

^∗∗∗^*p* < 0.01, ^∗∗^*p* < 0.05, ^*^p < 0.10.

Model 1 presents the basic logistic regression model, which only includes the control variables. Job position (i.e., manager) positively affects the likelihood that employees perceive a positive relationship between performance appraisal and IWB. In particular, as compared to employees in non-managerial positions, managers are more likely to perceive that the adoption of a performance appraisal system boosts their generation and implementation of creative ideas at the workplace (AME = 10%, *p* < 0.01 in Model 1; AME = 8%, *p* < 0.05 in Model 2). In contrast, age negatively affects the employees’ perception of performance appraisal as a booster of IWB. Employees aged 45–54 years old are less likely to perceive a positive impact of performance appraisal on IWB (AME = −14%, *p* < 0.05 in Model 1; AME = −12%, *p* < 0.05 in Model 2; AME = −12%, *p* < 0.05 in Model 3; AME = −11%, *p* < 0.05 in Model 4). Finally, Model 1 shows that working for manufacturing companies as well as in digitalized sectors or sectors with the potential to become digitalized have no statistically significant effect on the employees’ perception of performance appraisal as a booster of IWB. Moreover, this result holds on also in all subsequent models we estimated to test our hypotheses.

In Model 2, in order to test the main effects of the characteristics of performance appraisal on the dependent variable, we included, in addition to control variables, formal performance appraisal and the three performance appraisal criteria. The model shows that formal performance appraisal has a negative and significant effect on the employees’ perception that performance appraisal boosts IWB. Receiving a formal evaluation of one’s own performance decreases on average the likelihood that employees perceive a positive impact of performance appraisal on IWB by nearly 9 percentage points (*p* < 0.05). Therefore, Hypothesis 1 is not supported. In contrast, all three performance appraisal criteria considered in the present study have a positive and significant effect on the employees’ perception of performance appraisal as a booster of IWB. More specifically, exhibited skill-oriented appraisal increases the likelihood of such a perception by nearly 9% (*p* < 0.01), whereas result-oriented appraisal increases this probability by nearly 12% (*p* < 0.01). Thus, both hypothesis 2a and 2b are supported. Focusing on new competence-oriented appraisal, the positive effect becomes even stronger. Indeed, performance appraisal focused on the new competences developed by the employees while they performed their work increases the likelihood that performance appraisal is perceived as a booster of IWB by 15% (*p* < 0.01). This provides support for hypothesis 2c.

Models 3 and 4 test the presence of an interaction effect between the employees’ involvement in goal setting and, respectively, result-oriented, exhibited skill-oriented and new competence-oriented appraisals as predicted by hypotheses 3a, 3b, and 3c. First, we tested the direct effect of employees’ involvement in goal setting on the dependent variable (Model 3) and then we introduced the interaction terms (Model 4). In line with our expectations, we found a statistically significant, positive direct effect of employees’ involvement in goal setting on employees’ perception that performance appraisal boosts IWB (Model 3). Being involved in setting goals increases the likelihood of perceiving that performance appraisal relates positively to IWB by nearly 18% (*p* < 0.01). Finally, Models 4 shows that the interaction terms are not statistically significant. Thus, hypotheses 3a, 3b, and 3c are not supported.

## Discussion

The study draws on signaling theory and process-based approach to HRM (Bowen and Ostroff, 2004) to investigate which specific characteristics of performance appraisal are more likely to be perceived as promoting the generation and implementation of creative ideas at the workplace.

Our findings provide empirical evidence that some specific characteristics of performance appraisal (namely, the formality of the process and appraisal criteria, i.e., result- or competence-based criteria) may have a signaling function affecting the employees’ perception that performance appraisal has a positive impact on IWB.

More specifically, although we had hypothesized that in the employees’ perception the adoption of a formal performance appraisal system relates positively to IWB, our results show that the formality of performance appraisal actually reduces such a perception. In contrast with our expectations, this finding suggests that formality does not necessarily lead to a greater transparency and understandability, and thus to a better signaling effect, of the performance appraisal process. A possible explanation is provided by DeNisi and Sonesh (2011) who suggest that formality may result in an overemphasis on rating scales, appraisal form and other mechanisms aimed at reducing individual biases and improving rating accuracy, thus making it difficult to understand what is actually to be evaluated. In a similar vein, in the recent debate on performance appraisal, critics argue that formal appraisal systems fail to communicate what is important to the organization because over time they have reduced performance appraisal to a set of bureaucratic steps, procedures and tools of little value to leaders and employees (Pulakos et al., 2015; Cappelli and Tavis, 2016). Whereas some have thus suggested eliminating formal performance appraisal entirely (Culbert, 2010), others have argued for simple formal appraisal systems, considering that even when companies get rid of performance evaluations, evaluation is still done, but in a more subjective and non-transparent way (Pulakos and O’Leary, 2011; Goler et al., 2016). Regarding this, our study provides empirical evidence for those who recommend keeping formality to a minimum, by showing that the more performance appraisal relies on formal systems, the less it is likely to be perceived as a booster of IWB.

Moreover, our findings show that appraisal systems based on employees’ results affect positively the perception that performance appraisal boosts work-related innovation. This provides preliminary support for the argument that digitally transforming organizations should change their performance management systems, focusing more on pre-set quantitative results rather than on traditional criteria such as strictly, pre-defined behavior, time spent at the office and long working hours, in order to signal the greater emphasis on innovative work performance. On the other hand, even though in the employees’ perception result-oriented appraisal has a stronger positive impact on IWB than appraisal systems based on exhibited skills, we also found that such a perceived positive effect becomes even stronger when appraisal focuses on the new competences developed by employees. This result contributes to extant literature on employees’ perception of HRM practices to explain creativity and innovation in organizations and to the current debate about changes in performance appraisal enabled by digitalization of work, by showing that performance appraisal has a stronger signaling effect when it focuses on employees’ development rather than on how well they perform relative to a set standard of result or skill.

In line with previous studies, our findings also show the beneficial effect of employees’ involvement in goal setting (e.g., McConkie, 1982), particularly on employees’ creativity and innovation at work (de Jong and Den Hartog, 2007). However, in contrast with our expectations, we found no support for synergistic effects between employees’ involvement in goal setting and appraisal based on results, exhibited skills or new competences developed by employees. We can speculate that when employees evaluate a specific HRM practice, particularly performance appraisal and its effect on IWB, they will focus on the different features of such practice (e.g., appraisal criteria, the employee’s involvement) rather than on the whole practice and its set of features. This might question the complementarity or systemic approach suggested by extant literature on HRM bundles (for a recent review, see Boon et al., 2019). However, since this is definitely a preliminary result, further empirical examination is requested.

Finally, our study provides also evidence concerning the relationship between the sector and the sector’s level of digitalization, on the one hand, and the signaling effect of performance appraisal as a booster of IWB, on the other hand. Intriguingly, we found that such effect of performance appraisal is affected neither by the sector nor by the level of digitalization. Extant studies (e.g., Sanders and Yang, 2016; Bos-Nehles and Veenendaal, 2017) suggest that IWB are not encouraged in manufacturing firms since they are focused only on efficiency and the exploitation of extant knowledge. As a result, it is expected that manufacturing firms are less likely to use HRM practices to signal their emphasis on IWB. However, our study shows that also in manufacturing sectors employees may perceive performance appraisal as a booster of IWB and that such perception is affected by the specific characteristics of the appraisal system. Similarly, even though extant studies state that digitalization should boost innovative behavior, thus fostering the adoption of practices aimed at encouraging such behavior (e.g., Colbert et al., 2016; Schwarzmüller et al., 2018), we found that the signaling effect of performance appraisal as a booster of IWB is present also in lagging sectors and in sectors with the potential to become digitalized, depending on the specific characteristics of performance appraisal. We can thus suggest that also in lagging sectors and those with the potential to become digitalized innovation and IWB may be encouraged, probably with the aim to accelerate digitalization itself and that also in the digitalized sectors employees’ innovation at work, and specifically digitalization, may be further fostered by performance appraisal.

Taken together our findings also have managerial implications for organizations that aim to boost employees’ IWB so as to improve their ability to innovate in digital business environments. To begin, our results suggest that organizations should encourage informal practices of appraisal and feedback in order to consistently signal the above intent to their employees. In addition, being this study among the first to differentiate between different appraisal criteria, specific implications can be derived from the finding that competence-oriented appraisal as well as result-oriented appraisal positively affect the perception of performance appraisal as a booster of IWB. More specifically, in addition to what has been recommended so far, this study suggests to organizations to focus more on the employees’ new developed competences than on their achieved results or exhibited skills.

## Conclusion, Limitations, and Future Research Directions

The results of this study indicate that performance appraisal may play a strong signaling function indicating to employees the importance that their organizations place on IWB. More specifically, our study shows that as compared to informal feedback, formal performance appraisal reduces the perception of performance appraisal as a trigger of individual innovation at the workplace. In addition, in the employees’ perception, performance appraisal focused on the achievement of formal, individual performance targets affects IWB more positively than appraisal focused on pre-defined skills that employees exhibited as they performed their work. However, performance assessment focused on new competences developed by the employees has an even stronger effect than result-oriented appraisal. These results contribute to advance our understanding of how organizations should evaluate employees’ performance to capitalize on their IWB as a key to improve the organization’s ability to innovate in the digitalization era. Our study also suggests that performance appraisal that boosts employees’ innovation at work may be used both in the digitalized sectors to further foster digitalization, as well as in lagging sectors and those with the potential to become digitalized where enhancing IWB may be key to accelerate digitalization itself.

Despite the present study’s valuable contributions, it also has limitations that present some avenues for future research. First, we used a non-probabilistic sampling method in our data collection, and thus the convenience sample may be not representative of the larger working population in Italy. Therefore, we recommend employing randomly selected samples in future research. In addition, even though extant studies have extensively adopted self-reported measures of individual creativity since “employees themselves are best suited to report creativity because they are aware of the subtle things they do in their jobs that make them creative” (Dul et al., 2011, p. 723; see also Shalley et al., 2009; Baer, 2012), we recognize that the employees’ perception of performance appraisal as a booster of IWB may be biased by the personal definition of creativity and innovation. Moreover, we used a single item to operationalize the employees’ overall perception of performance appraisal as a booster of IWB. While some scholars have stressed that single-item measures offer the important advantages of being easier to understand than multi-item scales, not monotonous to complete and less time consuming, thus reducing response biases (Wanous et al., 1997), others tend to consider them as less appropriate for abstract constructs. Therefore, our findings ultimately need to be replicated by future research using multiple items to measure the employees’ overall perception of performance appraisal as a booster of IWB before they can be fully accepted.

## Data Availability

Restrictions apply to the dataset. The dataset for this manuscript will not be made publicly available because of privacy and ethical restrictions (it contains information that could compromise research participants’ privacy/consent). Requests to access the dataset should be directed to TF (tommaso.fabbri@unimore.it), member of the Marco Biagi Foundation Scientific Committee.

## Ethics Statement

In accordance with the authors’ Institution’s guidelines and national regulations, for this study an ethics approval was not needed. However, all the researchers have been subject to the Ethical Code of their affiliating Institution. Participation to this study was voluntary and withdrawal from the study was allowed at any given time. In compliance with the EU GDPR and Italian Legislative Decree no. 196 dated June, 30, 2003, all participants were written informed of the only scientific purposes of the research and their consent was implied through survey completion. Data were collected and used for the declared purposes and appropriate measures were applied to prevent the disclosure of information concerning individual persons and businesses.

## Author Contributions

YC, TF, and AS contributed to the conception and design of the study. SB organized the database and performed the statistical analysis. YC, TF, and AS wrote the sections “Introduction” and “Discussion”. YC and AS wrote the sections “Theoretical Framework,” “Procedure and Sample,” and “Conclusion, Limitations, and Future Research Directions”. YC and SB wrote the sections “Measures,” “Data Analysis,” and “Results”.

## Conflict of Interest Statement

The authors declare that the research was conducted in the absence of any commercial or financial relationships that could be construed as a potential conflict of interest.

## Footnotes

1 Sectors’ classification by level of digitalization is based on McKinsey Global Institute (2016) analysis of Europe’s digitalization on data collected in 15 European countries, including Italy. This analysis distinguishes among: (i) digitalized sectors, i.e., sectors that report high scores of overall digitalization since they are digitalized across all the key dimensions of digitalization: digital assets, digital usage and digital workforce; (ii) sectors with the potential to become digitalized, i.e., sectors with medium and low scores of overall digitalization but that have the potential to digitalize customer transactions or interactions and/or to provide digital tools to their workforce; and (iii) lagging sectors.
